# IMRT with Stereotactic Body Radiotherapy Boost for High Risk Malignant Salivary Gland Malignancies: A Case Series

**DOI:** 10.3389/fonc.2014.00268

**Published:** 2014-10-21

**Authors:** Sana D. Karam, Abdul Rashid, James W. Snider, Margaux Wooster, Shilpa Bhatia, Ann K. Jay, Kenneth Newkirk, Bruce Davidson, William K. Harter

**Affiliations:** ^1^Department of Radiation Oncology, The University of Colorado Denver, Aurora, CO, USA; ^2^Department of Radiation Oncology, Georgetown University Hospital, Washington, DC, USA; ^3^Department of Radiation Oncology, University of Maryland, Baltimore, MD, USA; ^4^Department of Radiology, Georgetown University Hospital, Washington, DC, USA; ^5^Department of Otolaryngology, Georgetown University Hospital, Washington, DC, USA

**Keywords:** parotid, SRS, SBRT, cyberknife, toxicity

## Abstract

Patients with high risk salivary gland malignancies are at increased risk of local failure. We present our institutional experience with dose escalation using hypofractionated stereotactic body radiotherapy (SBRT) in a subset of this rare disease. Over the course of 9 years, 10 patients presenting with skull base invasion, gross disease with one or more adverse features, or those treated with adjuvant radiation with three or more pathologic features were treated with intensity-modulated radiation therapy followed by hypofractionated SBRT boost. Patients presented with variable tumor histologies, and in all but one, the tumors were classified as poorly differentiated high grade. Four patients had gross disease, three had gross residual disease, three had skull base invasion, and two patients had rapidly recurrent disease (≤6 months) that had been previously treated with surgical resection. The median stereotactic radiosurgery boost dose was 17.5 Gy (range 10–30 Gy) given in a median of five fractions (range 3–6 fractions) for a total median cumulative dose of 81.2 Gy (range 73.2–95.6 Gy). The majority of the patients received platinum based concurrent chemotherapy with their radiation. At a median follow-up of 32 months (range 12–120) for all patients and 43 months for surviving patients (range 12–120), actuarial 3-year locoregional control, distant control, progression-free survival, and overall survival were 88, 81, 68, and 79%, respectively. Only one patient failed locally and two failed distantly. Serious late toxicity included graft ulceration in one patient and osteoradionecrosis in another patient, both of which underwent surgical reconstruction. Six patients developed fibrosis. In a subset of patients with salivary gland malignancies with skull base invasion, gross disease, or those treated adjuvantly with three or more adverse pathologic features, hypofractionated SBRT boost to intensity-modulated radiotherapy yields good local control rates and acceptable toxicity.

## Introduction

Salivary gland cancers are very rare subset of head and neck cancers with a diverse spectrum of histologic subtypes and natural history (1). For those eligible, surgery is the mainstay treatment with adjuvant radiation recommended in cases of high-grade histologies, advanced stage, and/or inadequate excision to maximize local control and overall survival (OS) (2–4). Patients whose tumors demonstrate base of skull invasion are known to have high rates of failure rates and stereotactic radiosurgery with gamma knife has been shown to significantly decrease the risk of failure (5). Similarly, for patients with unresectable disease higher failure rates have been reported (3). Although doses above 66 Gy have been shown to reduce rates of local failures, local control rates on the order of 30% have been reported in several series (3, 6).

In 2003, we began stereotactic body radiotherapy (SBRT) dose escalation for three subcategories of patients: those with skull base invasion, those treated definitively to gross disease with one or more adverse features, and those treated adjuvantly with three or more adverse pathological features. As salivary gland tumors are considered a radioresistant subset of head and neck cancers, hypofractionated dose escalation is well-suited based on the radiobiologic principle that the larger the fraction cell kill, the greater the probability of disease control (7). SBRT is particularly suitable for dose escalation due to the rapid radiotherapy dose fall-off with current image-guided radiotherapy technique. The CyberKnife SBRT system (Accuray, Inc., Sunnyvale, CA, USA) uses real-time image guidance for targeting to deliver highly conformal treatment of sites throughout the head and neck region. In this manuscript, we review our experience using SBRT boost with the Cyberknife system as a means for dose escalation for subsets of salivary gland tumors with high risk features.

## Materials and Methods

### Eligibility

After research ethics board approval was obtained, all patients with a diagnosis of salivary gland tumors treated with conventionally fractionated external beam radiotherapy followed by SBRT boost treated between 2003 and 2012 with definitive intent at our institution were retrospectively reviewed. Patients included were those who were inoperable and received treatment as definitive radiotherapy or received surgery for their primary tumor with macroscopic (R2) or microscopic (R1) residue and/or perineural or skull base invasion. The type and extent of surgery were dependent on the primary site, surgeon’s clinical judgment, and patient’s willingness to undergo resection. All except for one patient (no. 7 in Table 1) had histologically confirmed diagnosis of high-grade salivary gland malignancy, and all had available a history of present illness; a physical examination; diagnostic CT scans of the neck and chest; positron emission tomography computed tomography (PET/CT) imaging; and brain magnetic resonance imaging (MRI). Patients who had undergone prior radiotherapy were excluded. Charts were reviewed to determine patterns of disease failure, toxicity, and outcome. Before the treatment, patients’ cases were reviewed at the multidisciplinary head and neck tumor board. Radiosensitizing concomitant chemotherapy was administered at the discretion of the treating medical oncologist.

**Table 1:** **Initial patient characteristics and treatment results**.

No.	Age	Gender	Histology/site	Stage	SRS Vol.	Surgery	Reason for boost	IMRT dose/no. of fractions	SRS dose/no. of fraction	Cum dose	cCRT	POF	Time to failure	OS	Status
1	52	F	ACC/SM	T2N0M0	301.4	R1	PM, HG, SM origin	58.2/32	15.0/3	73.2	None	N/A	–	120	Alive, NED
2	68	F	PDC/P	T3N2M0	8.6	None	GD, HG	75.6/42	20.0/5	95.6	Carbo.	N/A	–	40	Alive, NED
3	47	M	Adenoca/P	T2N0M0	21.3	R1	PM, PNI, HG	63.0/35	11.25/5	74.3	None	N/A	–	46	Alive, NED
4	55	M	SCC/P	T4N2bM0	62.3	R1	SKI, HG	72.0/40	10.0/5	82.0	Cisplatin	N/A	–	82	Alive, NED
5	63	M	Adenoca/P	T4aN0M0	5.2	R2	GRD, SKI, HG	66.6/37	10.0/5	76.6	Carbo.	N/A	–	48	Alive, NED
6	80	F	SCC/P	T4N2bM0	457.0	None	GD, SKI, HG	50.0/25	25.0/5	75.0	Cetux.	N/A	–	12	Alive, NED
7	16	M	Adenoca/P	T2N0M0	230.0	R2	GRD, RR	63.0/35	21.0/3	84.0	None	N/A	–	22	Alive, NED
8	86	M	SCC/P	T2N2bM0	132.4	None	GR, HG	50.4/38	30.0/6	80.4	None	Local	15	23	DOD
9	51	M	Adenoca/P	T3N2bM0	626	R2	GRD, HG, RR	66.6/37	20.0/4	86.6	Carbo.	Distant	24	24	Alive with metastatic disease
10	66	M	Carc. Ex Pleo.	T4N3M0	460	None	GRD, SKI, CE	75.6/42	10.0/5	85.6	Carbo.	Distant	5	6	DOD

*The unit for dose is in Gy*.

*ACC, adenoid cystic carcinoma; Adenoca, adenocarcinoma; Carc. Ex Pleo, carcinoma ex pleomorphic; Carbo, carboplatin; CE, carotid encasement; Cis, cisplatin; Cetux, cetuximab; cCRT, concurrent chemoradiotherapy; Cum Dose, cumulative dose; DOD, died of disease; GD, gross disease; GRD, gross residual disease post resection; HG, high grade; NED, no evidence of disease; OS, overall survival; SM: SKI, skull base invasion; submandibular; PDC, poorly differentiated carcinoma; P, parotid; PM, positive margins; PNI, perineural invasion; POF, pattern of failure; RR, rapidly recurrent; SBRT Vol, stereotactic radiosrugery volume; SCC, squamous cell carcinoma*.

### IMRT treatment planning

Intensity-modulated radiotherapy (IMRT) treatment planning was delivered as previously described (8). Simulation CT, PET/CT, and pre- and post-operative MRI were fused for treatment planning purposes. GTV was contoured for any gross disease and the PTV consisted of GTV plus 1.5–2 cm margin. In cases of complete resection, the tumor bed was identified as the CTV and a 1–2 cm expansion to PTV was created. Elective nodal irradiation was at the discretion of the radiation oncologist, but generally followed the published criteria for estimating of risk of nodal involvement (4, 9). In post-operative cases, the treatment always started within 7–12 weeks from the date of surgery.

### Radiosurgery treatment planning

Planning for the radiosurgery boost began within 1 week of treatment. A fine cut (1.25 mm) CT was used for targeting and treatment planning. PET/CT and MRI fusion was done on all patients and used in defining the GTV. SBRT treatment planning was done as previously described, but adjustments were made by the treating physician based on tumor location and proximity of critical structures (10, 11). Three-dimensional non-coplanar beam arrangements were custom designed for each case to deliver highly conformal prescription dose distributions. Generally, more beams were used for larger lesions. As such, prescription lines covering the PTV were typically around 80% but ranging between 60 and 90% line rather than the more traditional 95–100%. Higher isodoses (“hotspots”) were manipulated to occur within the target and not in adjacent normal tissue. The following critical structures were contoured: spinal cord, brainstem, optic nerves, optic chiasm, orbits, lenses, cochlea, contralateral parotid, larynx, and brachial plexus (when the neck was included). As a general rule, prescription doses were dictated by tolerance of surrounding structures, which were in accordance with the AAPM Task Group 101 (12). BED was calculated for the IMRT and SBRT portions of the treatment using the formula [BED = *nd*(1 + *d*/α/β)], where *n* is the number of fractions and *d* is the dose per fraction, and using an α/β ratio of 10 for acute reacting tissues. The cumulative BED was established by combining the calculated BED of IMRT and SBRT.

### Toxicity evaluation and follow-up

Acute and late toxicity was defined according to the National Cancer Center Institute Common Terminology Criteria for Adverse Events, version 4.0, and determined by retrospective chart review. Acute toxicity was defined as occurring within 90 days of treatment completion. A complication that occurred during treatment that persisted after 90 days was also considered late toxicity. Regular follow-up is carried out 6 weeks post treatment, and every 2–3 months in the second year by the multimodality treatment team at our institution thereafter, and then in six monthly intervals including fibreoptic examination and imaging with MRI and/or PET/CT scan.

### Statistical analysis

Progression-free survival (PFS), locoregional control (LRC), and OS were defined as described (10). Disease response was assessed according to the response evaluation criteria in solid tumors (RECIST) (13). Survival outcomes were evaluated using the Kaplan Meier method. All analyses were performed in SAS version 9.2 (SAS Institute Inc., Cary, NC, USA).

## Results

### Patient and treatment characteristics

Patient characteristics and treatment results are shown in Table 1. The median age was 69 (16–86) with seven males and three females. All tumors were of parotid origin except for one submandibular tumor (no. 1 in Table 1). Patients presented with variable tumor histologies, and in all but one tumor, they were classified as poorly differentiated/high grade. Patient no. 7 had low grade classification, but he recurred 6 months after his first resection and had gross residual disease following his second resection. Four patients had gross disease (no. 2, 6, 8, 10), three had gross residual disease (no. 5, 7, 9), and three had skull base invasion (no. 5, 6, 10). Four patients presented with N_0_ disease, but in three of those (patients no. 1, 3, and 5 in Table 1) the nodes were electively included. Two patients (patients no. 7 and 9 in Table 1) had rapidly recurrent disease (≤6 months) that had been previously treated with surgical resection and underwent re-operation with radical resection followed by adjuvant radiation with SBRT boost. The rationale for SBRT boost is included in Table 1.

**Figure 1:**
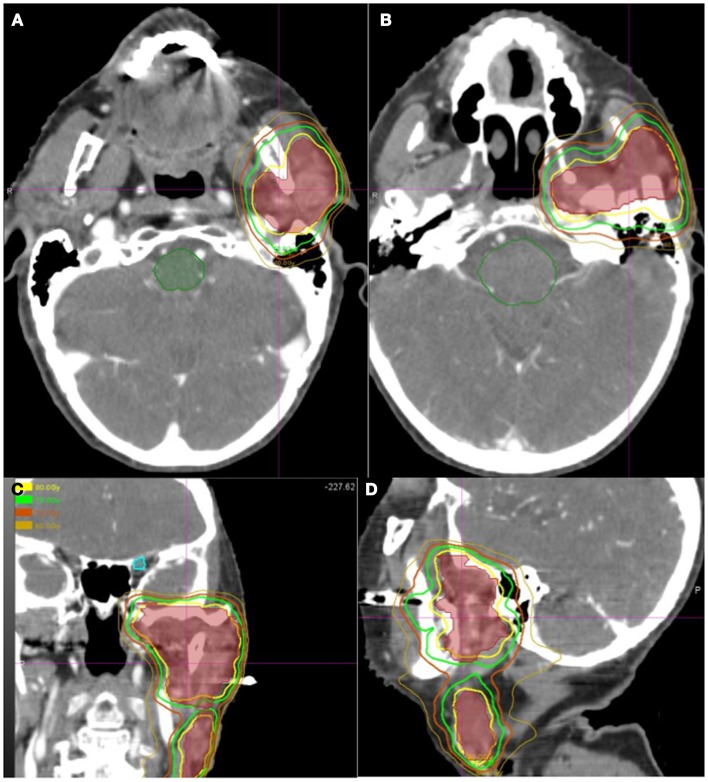
**Representative cumulative dose distribution of IMRT followed by SBRT plan of patient no. 6 of Table 1 who was treated definitively to gross disease with concurrent cetuximab for a Stage T4bN2b squamous cell carcinoma of the parotid**. **(A,B)** Representative axial views at the base of skull and the parotid gland. **(C,D)** Representative coronal and sagittal views. The outer brown isodose line represents the 70 Gy line, followed by the 75 Gy line in green, and 80 Gy in yellow. The innermost dark brown shaded area represents the planned treatment volume (PTV). The dark green contoured volume represents the brainstem.

**Figure 2:**
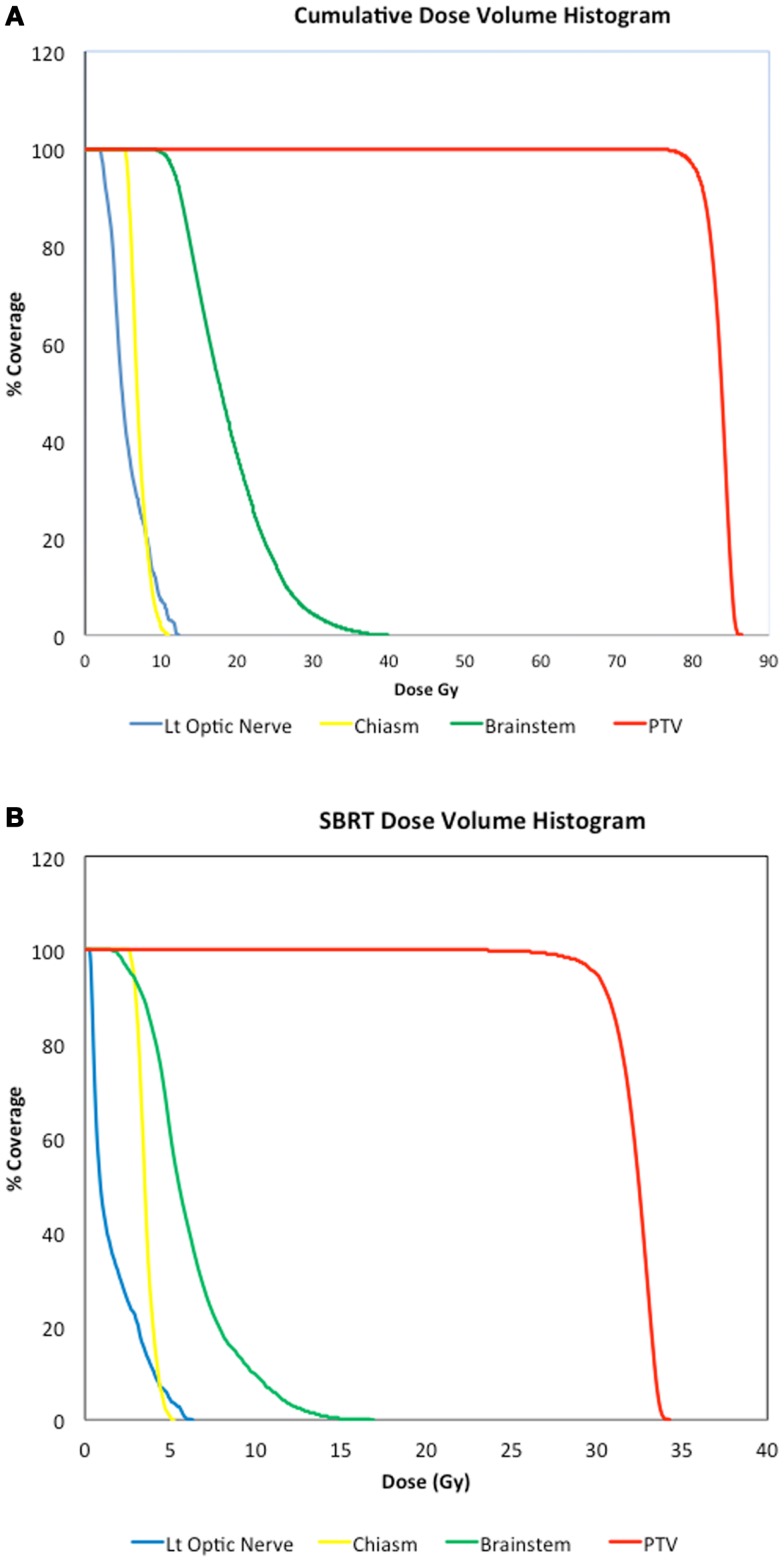
**(A)** Cumulative dose volume histograms (DVHs) of PTV and critical structures showing from left to right, PTV (red), brainstem (green), optic chiasm (yellow), and left optic nerve (blue). **(B)** SBRT DVHs for the corresponding structures shown in **(A)**. According to the TG 101 (12) SBRT tolerance for the brainstem is a maximum point dose of 25 Gy in five fractions and <5 cc of the brainstem receiving 6.6 Gy/fraction. The tolerance for the optic pathways is a maximum point dose of 25 Gy in five fractions.

All patients underwent IMRT followed by SBRT boost. Figures 1 and 2 show representative cumulative dose distribution and dose volume histograms (DVHs) of critical structures for an 80-year-old patient with SCC histology who underwent definitive RT with concurrent cetuximab for a Stage T4bN2b squamous cell carcinoma of the parotid. The median IMRT dose was 64.8 Gy (range 50–75.6) given in 1.8 Gy fractions in the majority of cases except one (patient no. 6), who received 2 Gy fractions. The median SBRT boost dosage per fraction was 4.5 Gy (range 2–7 Gy) given in a median of five fractions (range 3–6 fractions) for a total SBRT boost dose of 17.5 Gy (range 10–30 Gy) and a total median cumulative dose of 81.2 Gy (range 73.2–95.6 Gy). The median cumulative BED10 was 97.25 Gy (range 88.10–117.20 Gy) and the median cumulative BED3 was 143.8 Gy (range 120.5–167.6 Gy). The median interval between IMRT and initiation of SBRT boost was 1 week (range 0–2 weeks). The majority of the patients received concurrent chemotherapy with their radiation (Table 1). Carboplatin was the most administered chemotherapy, although cisplatin was used in one patient and Cetuximab in another.

### Survival outcomes and pattern of failures

**Figure 3:**
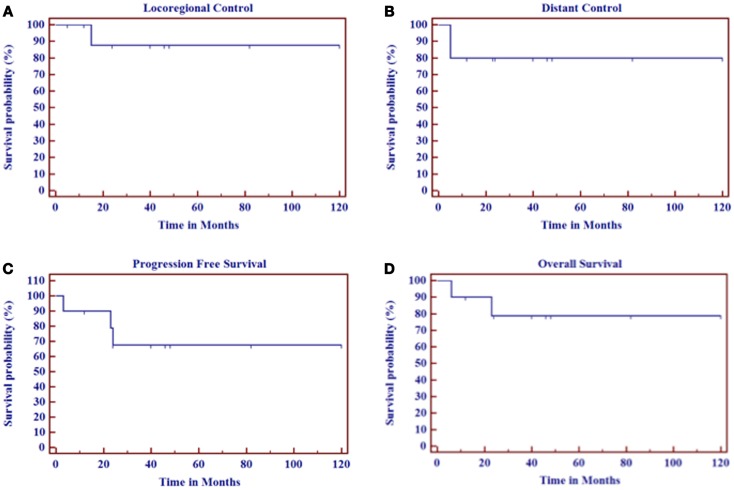
**Survival outcomes at a median follow-up of 29 months for all patients and 37 months for surviving patients**. **(A)** Locoregional control. **(B)** Distant control. **(C)** Progression-free survival. **(D)** Overall survival.

A summary of survival outcomes is shown in Table 2 and Figure 3. At a median follow-up of 29 months (range 12–120 months) for all patients and 43 months for surviving patients (range 12–120 months), actuarial LRC, distant control (DC), PFS, and OS were 88, 81, 68, and 79%, respectively (Table 2, Figure 2). Median values were not reached for any of the survival outcomes. Only one patient (no. 8 in Table 1) failed locally 15 months after the end of his radiation. He enrolled in phase I targeted biologic trials with local progression of his disease and died 8 months later. Two patients (no. 9 and 10 in Table 1) developed distant metastases. One died of disease progression and the other continues to receive palliative radiation therapy and systemic chemotherapy/biologics.

**Table 2:** **Crude survival outcomes**.

Median follow-up in months (range)	29 (5–115)
Median follow-up surviving patients in months (range)	37 (6–115)
Actuarial 3-year locoregional control (failed/controlled)	88% (1/9)
Actuarial 3-year progression-free survival (failed/controlled)	64% (3/7)
Actuarial 3-year overall survival (dead/alive)	77% (2/8)
Actuarial 3-year distant control (failed/controlled)	80% (2/8)

*Median values were not reached for any of the survival outcomes*.

### Toxicity

Acute toxicities included mucositis, dysguesia, skin toxicity, and odynophagia. Generally, the acute symptoms were managed with palliative treatments such as skin creams, mouth wash, narcotics, and nutritional support. There were no grade IV or V toxicities or unexpected acute toxicities. None of the patient required feeding tube placement, and there were no treatment related deaths. Long-term toxicity included hypothyroidism in two patients (no. 1 and 3 in Table 1), sensorineural hearing loss on the affected side in four patients (no. 2, 3, 5, and 6 in Table 1), overnight xerostomia in three patients (no. 3, 4, and 5 in Table 1), and fibrosis in six patients (no. 3, 4, 5, 7, 8, and 9 in Table 1), which was managed with physical therapy in all patients. One patient developed osteoradionecrosis (no. 4 in Table 1), which was managed with hyperbaric oxygen and surgical reconstruction. One patient (no. 3 in Table 1) developed graft ulceration that was surgically reconstructed. Both remain without evidence of disease at their last follow-up. There were no patients with facial nerve injuries.

## Discussion

In this manuscript, we presented our institutional experience with subsets of high risk salivary gland tumors with a high propensity for local failure (3, 6, 9). This included those with skull base invasion, those treated definitively to gross disease with one or more adverse features, and those treated adjuvantly with three or more adverse pathological features. Although these tumors are relatively rare, the radioresistant nature of these tumors and challenging location in extremely critical areas in the head and neck make them an ideal target for dose escalation with stereotactic radiosurgery boost. Severe hypofractionation such as permitted by SBRT-like dose-boosting is also thought to radiobiologically counterbalance the loss of reoxygenation within a few fractions (14).

Douglas et al. showed that for patients with salivary gland neoplasms involving the base of skull, treatment with GammaKnife stereotactic radiosurgery boost, following neutron radiotherapy, improved local control rates to about 80% at 40 months compared to historical controls receiving neutron therapy alone (5). Recent results in dose escalation using particle therapy have been reported (15–17). Pommier et al. treated 23 patients with adenoid cystic carcinoma and skull base invasion with proton dose escalation to 75.9 Gy (median) in various fractionation schemes with a reported 5-year LRC rate of 93% (17). Dose escalation with carbon ion therapy in a Japanese series including a variety of head and neck cancers – eight salivary gland tumors of different histologies – showed 100% local control rate at 5 years (16). Most recently, interim analysis of the German COSMIC trial using dose escalation with carbon ion boost for salivary gland malignancies showed tolerable acute toxicities, but the follow-up was too short for assessment of survival outcomes or late toxicity (15).

Our results show good local control data comparable to that achieved by Gammaknife radiosurger, although our sample included more patients with unresectable disease and gross residual disease (5). The acute and long-term toxicity encountered in our patient population was clearly tolerable and comparable to other treatment modalities (5, 17, 18). The small sample size and the retrospective nature of the study prohibit us from performing any correlational analysis for prognosticators of late toxicity. In our previously published reirradiation series, we reported a correlation between cumulative doses over 90 Gy and the development of soft tissue necrosis (10). In the RTOG-MRC randomized trial that showed 67% LRC compared to 13% in patients with unresectable disease, significant toxicities such as temporal lobe necrosis, ulceration, and spinal cord myelopathy were also reported (18). Although Gammaknife dose escalation following neutron treatment for patients with skull base invasion did not result in increased toxicity compared to that of neutron alone, there was significant toxicity associated with neutron treatment overall (5). In the proton dose escalation for patients with adenoid cystic carcinomas, one patient developed temporal lobe necrosis (17).

Our DC8 rates also show good results. This could be attributed to the fact that the majority of our patients received concurrent chemotherapy. Tanvetyanon et al. has shown that for locally advanced or high-grade salivary gland tumors, the addition of concurrent chemotherapy to post-operative radiation treatment significantly improves survival outcomes (19). The RTOG 1008 is currently enrolling patients to address this question.

## Conclusion

The results of our case series show that, at 3-year follow-up, dose escalation with SBRT following IMRT results in good local control for patients with high risk salivary gland malignancies. Further follow-up is needed as late disease recurrence is not uncommon for these tumors (20). Our results are also limited by the small sample size, retrospective nature of the study, and heterogeneity of the patient population and treatment parameters.

## Conflict of Interest Statement

The authors declare that the research was conducted in the absence of any commercial or financial relationships that could be construed as a potential conflict of interest.

## Abbreviations

DC, distant control; DVH, dose volume histogram; IMRT, intensity-modulated radiotherapy; LRC, locoregional control; MRI, magnetic resonance imaging; OS, overall survival; PET/CT, positron emission tomography/computed tomography; PFS, progression-free survival; RECIST, response evaluation criteria in solid tumors; SBRT, stereotactic body radiotherapy; SRS, stereotactic radiosurgery.
